# An integrated strategy of knowledge application for optimal e-health implementation: A multi-method study protocol

**DOI:** 10.1186/1472-6947-8-17

**Published:** 2008-04-24

**Authors:** Marie-Pierre Gagnon, France Légaré, Jean-Paul Fortin, Lise Lamothe, Michel Labrecque, Julie Duplantie

**Affiliations:** 1 Research Center of the Centre Hospitalier Universitaire de Québec, Québec, Canada; 2 Department of Nursing, Université Laval, Québec, Canada; 3 Department of Family Medicine, Université Laval, Québec, Canada; 4 Department of Social and Preventive Medicine, Université Laval, Québec, Canada; 5 University Affiliated Centre of the Centre Santé et Services Sociaux de la Vieille Capitale, Québec, Canada; 6 Department of Health Management, Université de Montréal, Montréal, Canada

## Abstract

**Background:**

E-health is increasingly valued for supporting: 1) access to quality health care services for all citizens; 2) information flow and exchange; 3) integrated health care services and 4) interprofessional collaboration. Nevertheless, several questions remain on the factors allowing an optimal integration of e-health in health care policies, organisations and practices. An evidence-based integrated strategy would maximise the efficacy and efficiency of e-health implementation. However, decisions regarding e-health applications are usually not evidence-based, which can lead to a sub-optimal use of these technologies. This study aims at understanding factors influencing the application of scientific knowledge for an optimal implementation of e-health in the health care system.

**Methods:**

A three-year multi-method study is being conducted in the Province of Quebec (Canada). Decision-making at each decisional level (political, organisational and clinical) are analysed based on specific approaches. At the political level, critical incidents analysis is being used. This method will identify how decisions regarding the implementation of e-health could be influenced or not by scientific knowledge. Then, interviews with key-decision-makers will look at how knowledge was actually used to support their decisions, and what factors influenced its use. At the organisational level, e-health projects are being analysed as case studies in order to explore the use of scientific knowledge to support decision-making during the implementation of the technology. Interviews with promoters, managers and clinicians will be carried out in order to identify factors influencing the production and application of scientific knowledge. At the clinical level, questionnaires are being distributed to clinicians involved in e-health projects in order to analyse factors influencing knowledge application in their decision-making. Finally, a triangulation of the results will be done using mixed methodologies to allow a transversal analysis of the results at each of the decisional levels.

**Results:**

This study will identify factors influencing the use of scientific evidence and other types of knowledge by decision-makers involved in planning, financing, implementing and evaluating e-health projects.

**Conclusion:**

These results will be highly relevant to inform decision-makers who wish to optimise the implementation of e-health in the Quebec health care system. This study is extremely relevant given the context of major transformations in the health care system where e-health becomes a must.

## Background

Effectiveness, efficiency and equity are the core goals of the Canadian health care system [1]. To achieve these goals, several experiments based on e-health – the application of information and communication technologies (ICT) as tools to support health care[2] – have been implemented. In spite of limited and sometimes conflicting evidence on the effects of e-health applications, ICT are increasingly regarded as promising tools for supporting access to quality health care services, information flow, integration of health care services, and interprofessional collaboration [3-5]. Consequently, evaluating the impact of e-health applications at various levels constitutes an essential step to understand their associated risks and benefits. In turn, the production and utilisation of scientific evidence is essential in order to legitimate e-health investments [6-8]. It is thus essential that e-health applications are rigorously evaluated for their effectiveness, efficiency and impact on equity before promoting their widespread dissemination in the Canadian health care system [9].

### E-health in the Canadian health care context

E-health gathers two main categories of applications: telehealth and electronic health record (EHR) [2]. In Canada, the implementation and the diffusion of telehealth and EHR in the health care system is a priority for the next years [10]. These technologies promise positive impacts for patients, health care professionals, health care organisations, as well as for the health care system and the population as a whole [3,4]. However, several questions remain with respect to the potential implications of e-health on safety and quality of health care services, security and confidentiality of data, and costs [8,11]. Hence, in order to avoid expensive pitfalls, an optimal implementation of e-health based on the best level of evidence is desirable.

The introduction of e-health applications is often carried out in a context of uncertainty where several alternatives are possible since reasonable evidence on the effectiveness, efficiency and costs of the technology is not available. In this context, the term 'optimal use' refers to a decision which maximises the benefits and minimises the risks by taking into account alternative options, costs and resources available, as well as values and preferences of patients and health care providers [12]. In this study, an optimal integration of e-health applications is conceived as one that considers the best scientific knowledge available, as well as the specific context and the values of the different groups of stakeholders involved.

### Knowledge utilisation in health care decision-making

The application of scientific evidence in the development of health care policies and practices remains limited despite extensive efforts invested over the last decades [7,13]. As so, the adoption and diffusion of health technologies and innovations is often influenced by factors other than scientific, which can involve adverse consequences for the health care system [14,15]. The effectiveness of strategies aimed at the integration of the best scientific evidence in professional practices has been extensively reviewed [16,17]. However, most of previous research on the use of evidence in health care policies and practices were not based on theoretical foundations. This constitutes an important limit since theories and models are essential for a systematic analysis of the factors influencing the utilisation of evidence in clinical, organisational and policy decisions [18,19]. Furthermore, factors influencing the utilisation of evidence to support decision-making regarding e-health implementation are still unknown [20]. Decision-makers need evidence on the effectiveness of e-health applications, but also on the conditions allowing their applicability in specifics contexts [21]. This study will likely contribute to fill these gaps.

### Assessment of e-health applications

The assessment of health technologies must be based on rigorous methods to produce evidence in order to support decision-making [7,22,23]. However, several studies[7,24-26] question the systematic application of traditional methods to e-health assessment and propose strategies adapted to the specific characteristics of e-health technologies. As so, the assessment of e-health applications should not only examine the effects of these new technologies on quality, accessibility and services costs, but should also seize the interactions between technical equipment and infrastructure, humans, and the socio-political context [27]. Decisions regarding e-health implementation are thus likely to be influenced by various types of knowledge and elements of context that are important to consider for an optimal integration of these technologies in the health care system [26].

### Goal and objectives

Given the lack of evidence on how to effectively implement e-health applications in complex health care systems, it is imperative to improve knowledge on decision-making processes regarding the integration of e-health. This study aims at understanding factors influencing the application of scientific knowledge for an optimal implementation of e-health in the health care system. To do so, decision-making processes are being analysed at three decisional levels: 1) health policies (*macro*); 2) health care organisations (*meso*); 3) professional practices (*micro*).

The project is structured around four specific objectives: 1. To explore the utilisation – or non-utilisation – by decision-makers from each level (*macro*, *meso *and *micro*) of various sources of knowledge, including scientific evidence, to support e-health implementation; 2. To analyse factors determining the application of scientific evidence to support e-health implementation; 3. To analyse interactions between the three decisional levels that influence the application of scientific evidence in e-health decision-making; 4. To identify key elements in order to propose an integrated strategy for an optimal e-health implementation based on scientific evidence and the specific context.

### Theoretical frameworks

Decisions regarding the implementation of e-health application in the health care system occur at three decisional levels: political, organisational and clinical [28]. Specific determinants can influence the degree of knowledge utilisation in the decisions process at each level [24]. Thus, it seems essential to consider these decisional levels in a concomitant way in order to better understand the complex phenomenon than is the integration of a e-health in the health care system [29].

Furthermore, decisions made at each level are interdependent since a decision made at one level can impact on the other levels. For instance, the decision to invest in a particular technology made at the health policy level could influence resources allocation at the organisational level and health care professionals' involvement at the clinical level [15,30]. A multidimensional analysis is thus necessary in order to draw an overall picture of the factors influencing knowledge application to support the implementation of e-health.

**Figure 1:**
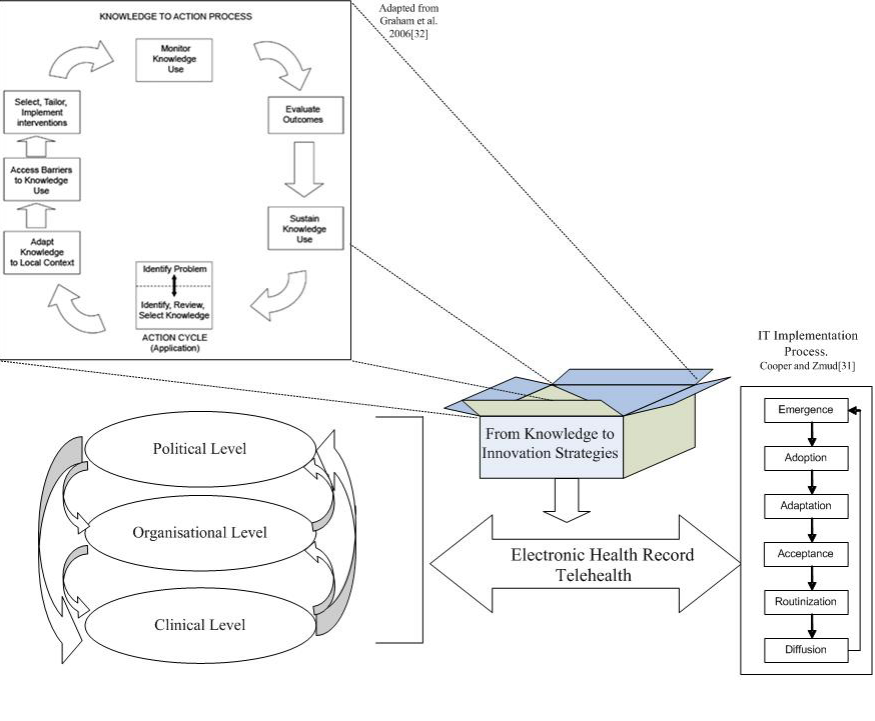
Integrated conceptual framework of e-health decision-making processes.

Figure 1 presents an integrated conceptual framework of e-health decision-making processes at the various decisional levels and schematises the cycle of knowledge utilisation. This framework is inspired by various models [31,32] and identifies principle factors influencing knowledge utilisation in decision-making processes. The phases of knowledge application used it this framework are borrowed from Graham et al. 2006 [32]: (1) identify problems and relevant knowledge; (2) adapt knowledge; (3) assess barriers to knowledge use and factors facilitating it; (4) implement interventions; (5) monitor knowledge use; (6) evaluate outcomes; (7) sustain knowledge use [32]. The application of knowledge to support decision-making on e-health is thus conceptualised as being the 'black box' of the interactions between the knowledge available and the decisions made at each decisional level at the various phases of e-health implementation. Based on the IT implementation process of Cooper and Zmud [31], the phases of e-health implementation are: emergence, adoption, adaptation, acceptance, routinisation, and diffusion.

Furthermore, specific theoretical frameworks pertain to decision-making processes at each level. They are described for each level in the Method section.

## Methods

### Study design

A multiple case study is being conducted to gain a comprehensive understanding of the decision-making processes regarding the implementation of the e-health applications selected. Case study appears as an appropriate strategy for this study since it makes it possible to consider a complex phenomenon within its social, political and historical context [33]. This strategy has been successfully applied in similar studies in the field of research utilisation in health care decision-making[34] and diffusion of health technologies [15]. Case study allows an in-depth analysis of the dynamics involved in the utilisation of knowledge in decisions regarding the implementation of e-health applications. Moreover, this design allows inter-cases comparisons which will contribute to the rigour of the analyses while making it possible to confirm assumptions or to propose alternative explanations to the phenomenon [33].

### Setting and e-health applications

This multiple case study takes place in the Province of Quebec and focuses on two specific applications: telehealth and electronic health record. These applications have been selected based on their key position in current federal policies as regards computerisation of the health care system in Canada [10] as well as their importance at the provincial level in Quebec [35]. Specific methods and strategies are being used to explore factors influencing the utilisation of scientific knowledge to support e-health implementation at each decisional level.

#### Political level

A critical incidents analysis will be conducted to identify key decisions that have influenced the implementation of telehealth and EHR in the Quebec health care system. This method has been used in previous studies on the impact of health technology assessment on political decisions [13,22]. It consists in identifying, through document analysis and/or contact networks, the 'incidents' that represent specific events or decisions having affected the implementation of the technology. A summary of these critical incidents will be prepared and validated among a purposive sample of key stakeholders who have been involved in these critical incidents.

Then, factors that supported or constrained utilisation of scientific knowledge in the decisions identified will be explored through interviews with key stakeholders who have been involved in these decisions. A semi-structured interview guide will be used to explore specific questions that will be adapted to the nature of the decision and the position of the interviewed stakeholder. Approximately 40 keys informants, including policy-makers, e-health projects managers, evaluators and representatives of various lobbies (professional associations, technological companies, etc.) will be interviewed. Nevertheless, this number may vary according to the data saturation criteria [36]. Interviews are planned to last about one hour and will be audio recorded after obtaining participant's consent.

At the political level, decisions regarding the implementation of telehealth and EHR are analysed according to three principle themes: 1) funding, diffusion and sustainability of e-health projects, 2) evaluation of e-health projects and its impact on decision-making; 3) other sources that have influenced decisions regarding the implementation of e-health applications. The characteristics and position of stakeholders will be analysed based on Monnier's classification [37], and the creation of coalitions and alliances will be studied based on the frameworks of Gamson [38] and Lemieux [39]. These frameworks will allow identifying stakeholders who have been involved in important decisions regarding e-health implementation and how their specific position and characteristics have influenced knowledge utilisation in these decisions.

The N*Vivo software will be used to perform qualitative analyses. A thematic content analysis will be carried out according to the method described by Huberman and Miles [40] which implies coding, organizing and linking the material collected from interviews. The codification will be based on concepts from theoretical frameworks relevant to health care policy analysis [37-39]. To ensure the internal validity of the analytical process, codification of the interviews will be made independently by two researchers of the team. The results will then be shared and consensus will be sought for final codification. Interview material will be categorised using this codification tree. Constant comparison and iterations methods will be used to identify the factors that have facilitated or constrained the utilisation of knowledge to support decisions surrounding e-health implementation according to stakeholders' characteristics and role.

#### Organisational level

At the organisational level, one telehealth and one EHR project implemented since at least one year will be selected for the case study. For telehealth, a telehomecare project in Gaspésie – Îles-de-la-Madeleine, has already be identified. A primary care clinic that has implemented an EHR has also agreed to participate.

Semi-structured interviews with key decision makers (regional and local decision makers, project managers and health care professionals), and document analysis will be used for data collection. Participants will be identified through the contacts network method[36] and will then be invited to take part to the study. A total of 20 interviews is planned (10 for each project), but this number could change according to the data saturation criteria [36]. A major element to consider in selecting participants will be the representation of all points of view, namely the presence of decision-makers from the various stakeholders groups involved in the project (professionals, managers, promoters, evaluators). Interviews – of approximately one hour duration – will be audio recorded with participants' consent.

Theoretical models from organisation sciences, such as the neo-institutional theory [41], the theory of communities of practice [42] as well as professional theories [43,44] provide a framework to examine the contribution of knowledge in decision-making pertaining to e-health projects. The neo-institutional theory proposes the concept of isomorphism, according to which the borders between the organisations become less and less rigid because of associations that are formed between members of various organisations. This concept is highly relevant given the networking purposes of e-health applications. Furthermore, the addition of the concepts of 'communities of practice' and 'occupational boundaries' to the institutional theory, as suggested by Aanestad and al [45], allows conceiving knowledge utilisation in decisions on e-health as a phenomenon of networking between various organisations and professional groups. These concepts will be used to analyse the application of knowledge in organisational decision-making during the implementation of e-health projects.

Data collected will be analysed qualitatively using the N*Vivo software. An iterative analytical method will be employed starting with concepts found in the theoretical models selected [41-45]. These categories could be adjusted through interactions with the field. The first interviews will be independently coded by two researchers of the team and will be discussed with other members of the team in order to reach a consensus on a final codification tree. Moreover, a participative approach encouraging feedback from representatives of each project will ensure the validity of the analyses [34] and is likely to favour a greater appropriation of the results by the stakeholders [46].

#### Clinical level

At the clinical level, the psychosocial determinants of health care professionals' intention to use e-health in their practice are explored using the Theory of Interpersonal Behaviours (TIB) [47]. The TIB is considered as an exhaustive psychosocial theory since it integrates most of the construct found in other theories as well as dimensions such as values, social roles and norms that are not taken into account in other models. Moreover, the TIB regards the culture or the subculture as a factor influencing behaviour [48]. According to this theory, human behaviour is formed by three components: intention, facilitating conditions, and habit. *Intention *refers to the individual's motivation regarding the performance of a given behaviour. *Facilitating conditions *represent objective factors that can make the realisation of a given behaviour easy to do. *Habit *constitutes the level of routinisation of a given behaviour, i.e. the frequency of its occurrence.

In the TIB, the behavioural intention is formed by attitudinal as well as normative beliefs. Attitudinal beliefs comprise affect and perceived consequences. *Affect *represents an emotional state that the performance of a given behaviour evokes for an individual. It is considered as the affective perceived consequences of the behaviour, whereas *perceived consequences *refer to the evaluation by the individual of the possible consequences of the behaviour. The TIB also distinguishes between two normative dimensions: social and personal. Perceived social norms are formed by normative and role beliefs. *Normative beliefs *consist of the internalisation by an individual of referent people or groups' opinion about the realisation of the behaviour, whereas *role beliefs *reflect the extent to which an individual thinks someone of his or her age, gender and social position should or should not behave. With respect to the other normative components of the TIB, the *personal normative belief *represents the feeling of personal obligation regarding the performance of a given behaviour, whereas *self-identity *refers to the degree of congruence between the individual's perception of self and the characteristics associated with the realisation of the behaviour. Previous work on clinicians adoption of health care technologies [49] confirm the utility of the TIB to understand the behaviours of health care professionals.

Based on results from previous work [49] and on our recently completed Cochrane systematic review on "*Interventions for promoting information and communication technologies adoption in healthcare professionals*" [50], a questionnaire will be developed. The questionnaire will be built according to a consensus of experts in social psychology [51] and will follow the methodology recommended by Gagné and Godin [52] This questionnaire will assess the psychosocial factors influencing the adoption of telehealth and EHR by health care professionals. It will also assess the role of scientific evidence to support clinicians' decision-making regarding their use of these e-health applications.

The etic-emic approach [53] will be used in the development of the questionnaire since it allows adapting the theoretical constructs (etic dimension) to specific context of the culture studied (emic dimension) according to the studied population. A convenient sample of about 20 clinicians will be selected to complete an open-ended questionnaire. Qualitative analyses will be carried out in order to extract participants' beliefs corresponding to each of the TIB constructs. The frequency of each belief will be compiled in order to identify the modal salient beliefs in the population. Identified beliefs will then be used as question items for measuring each of the theoretical constructs in the study questionnaire. The internal consistency of theoretical constructs and the temporal stability of their measurement [54,55] will be checked using the test-retest method by asking 30 clinicians to complete the questionnaire twice within a two-weeks interval. A final version of the questionnaire will be prepared following the test-retest.

The sample size necessary depends on the number of theoretical variables (7) and studied external variables (5). According to Cohen [56], a sample of 547 participant is necessary to perceive the effect of two groups of variables in the regression of the dependent variable: the first formed group of the seven variables of the TIB (R^2 ^equal to 0.10); the second group made up of five additional variables with an increase in R^2 ^equalizes to 0.02 (alpha = 0.05 and power of 0.80 for the two groups of variables). Thus, by estimating the participation rate at 40%, which corresponds to the average found in similar studies [57], it will be necessary to target 1368 physicians in order to recruit 547 of them. Strategies aiming at increasing response rate will be used such as the participation of the Head of Department and sending recall letters according to the procedure suggested by Dillman [58].

The following analyses will be carried out using the SAS software: 1) distribution of the variables in terms of percentage and average; 2) Pearson or Spearman coefficients of correlation; 3) multiple regression of the factors (independent variables) predicting clinicians' intention to use evidence on e-health (dependent variable). The independent variables that will be evaluated are: affect, perceived consequences, normative beliefs, role beliefs, personal normative belief, self-identity, and facilitating conditions; 4) the influence of the external variables (sociodemographic and professional) on the intention by comparing R^2 ^of the model including all variables to the model containing only the psychosocial variables [59]; and 5) a multiple variance analysis in order to determine the theoretical constructs allowing to distinguish the subjects intending or not to use knowledge to support their decisions.

### Triangulation and integration of the results

This project is highly innovative since it proposes a multidimensional analysis of the factors influencing the utilisation of knowledge in decision-making on e-health implementation at three decisional levels. Triangulation of the results, theories, and methods [60] will allow considering a whole set of factors that could influence the application of knowledge across three decisional levels and identifying differences and similarities between those. The combination of qualitative and quantitative approaches allows exploring the introduction of e-health under different angles, which will contribute to a deeper understanding of this innovative phenomenon [61].

Results triangulation is based on the integrated conceptual framework (Figure 1). Matrices will be used to classify the factors associated with each component of the knowledge application cycle (identification; adaptation; obstacles; intervention; follow-up; evaluation; maintenance) and that, for each decisional level and each technology considered (telehealth and EHR). A transversal analysis will allow comparing the mechanisms involved in knowledge application for each technology. As such, we will be able to identify differences and similarities between factors influencing knowledge application according to the specific context of the technology implemented. Particular attention will be given to factors influencing the process of normalisation, that is the potential for complex interventions to become routinely embedded in everyday practices [62]. The conditions necessary to support the introduction of complex interventions such as e-health applications and the factors that promote or inhibit their success and failure in practice will thus be explored through the normalisation process model, developed by May [62].

### Ethical considerations

All data collected for the document analysis in this study will be obtained from publicly available sources. Participants to individual interviews or questionnaire survey will be given specific consent forms presenting research objectives and information about research implications. Ethics approval for the study protocol has been received from the Research Ethics Board of the Centre Hospitalier Universitaire de Québec (approved August 8 2007; ethics number 5-06-09-02)

## Discussion and implication

In order to foster evidence-based decisions for e-health implementation, it is important to consider simultaneously two components that are the definition of what constitutes evidence and the process of decision-making in itself [28]. The definition of a evidence is strongly influenced by the various issues that are present in the specific context [28]. These issues vary according to the decisional level involved and the stage of project's development [63]. The introduction of e-health often begin with pilot projects that are of restricted duration, which limits the production of scientific evidence and its integration to the development of e-health applications in the health care system [62,64]. The proposed study is relevant given the current lack of knowledge concerning the introduction of e-health applications and their impacts. In this context of uncertainty, it is essential to invest in applications that have proven their effectiveness, while taking into account the specific context in which they are introduced. This study will contribute to identify gaps in terms of knowledge production and utilisation in order to ensure an optimal implementation of e-health solutions based on scientific evidence.

Knowing the processes influencing knowledge application in e-health decisions is central in order to contribute to a planned integration of these technologies into the health care system. Thus, this research supports the development of a planned strategy of e-health implementation supported by empirical and theoretical knowledge, while taking into account the context of decision-making (evidence-based, theory-driven, and contextualised). This research is being conducted in close collaboration with decision-makers from the Quebec's Ministry of Health which favours knowledge sharing and its application to support decisions in real life context. As so, our results are likely to be used in order to inform decision-makers about strategies for an optimal implementation of e-health in the Quebec health care system. This study is extremely relevant given the context of major transformations in the health care system where e-health becomes a must. The innovative character of this research as well as its strong theoretical and empirical foundations is likely to open on the development of new practices for the production and application of knowledge as regards the development of e-health solutions for an equitable, effective and efficient health care system.

## Competing interests

The authors declare that they have no competing interests.

## Authors' contributions

All authors collectively drafted the research protocol and approved the final manuscript. MPG is its guarantor.

## Pre-publication history

The pre-publication history for this paper can be accessed here:


